# Supplementary material for: Knockout of stim2a Increases Calcium Oscillations in Neurons and Induces Hyperactive-Like Phenotype in Zebrafish Larvae
Source: Int J Mol Sci. 2020 Aug 27;21(17):6198. doi: 10.3390/ijms21176198 (PMC7503814; doi:10.3390/ijms21176198)
Supplement: Supplementary file 1 [file ijms-21-06198-s001.zip › Supplementary information.docx]

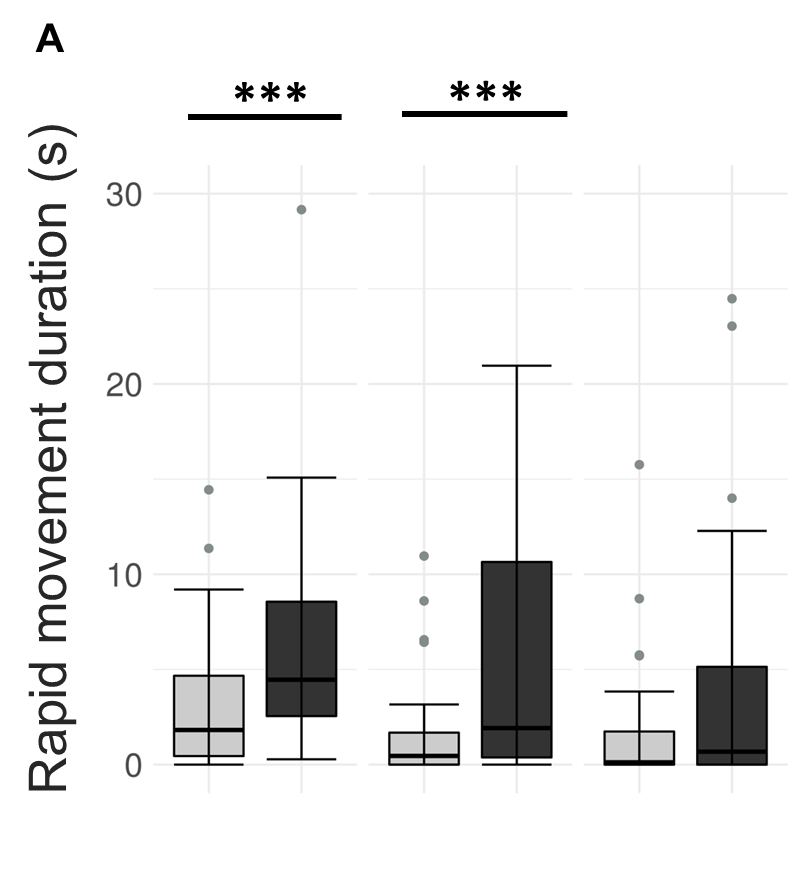

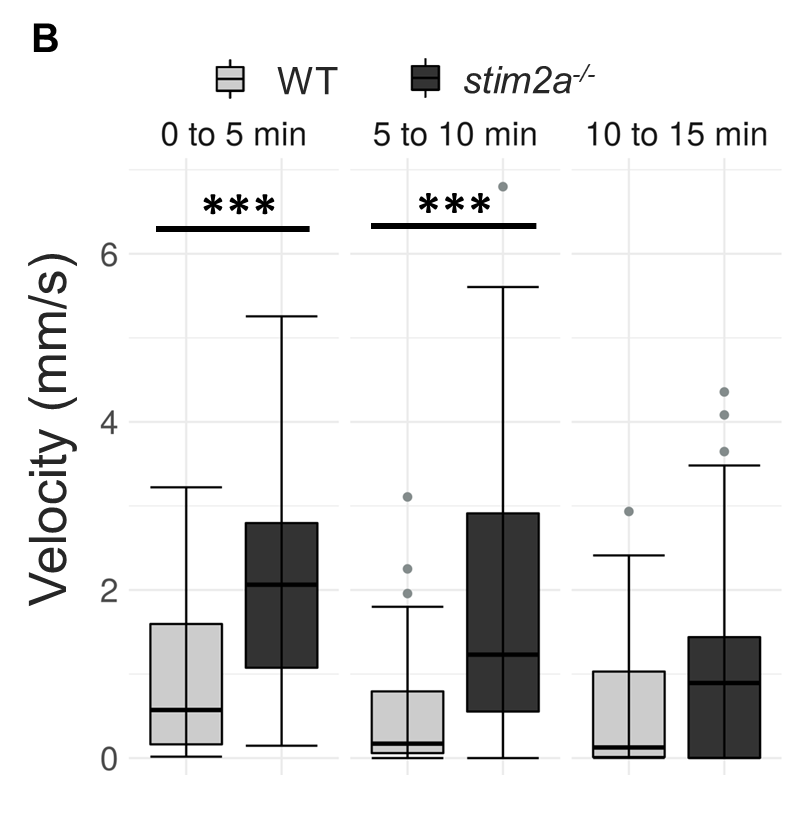

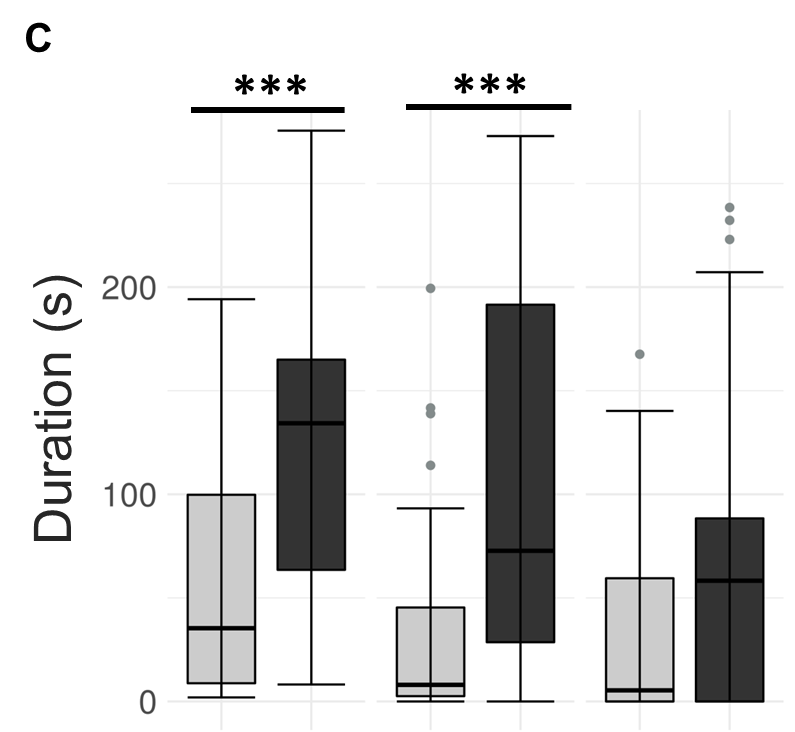


**Fig. S1.** **Open field test adopted to zebrafish larvae thorough analysis:** The detailed analysis of other parameters, such as (A) velocity, (B) time spent moving and (C) time spent moving with a high speed. (****p* < 0.001)


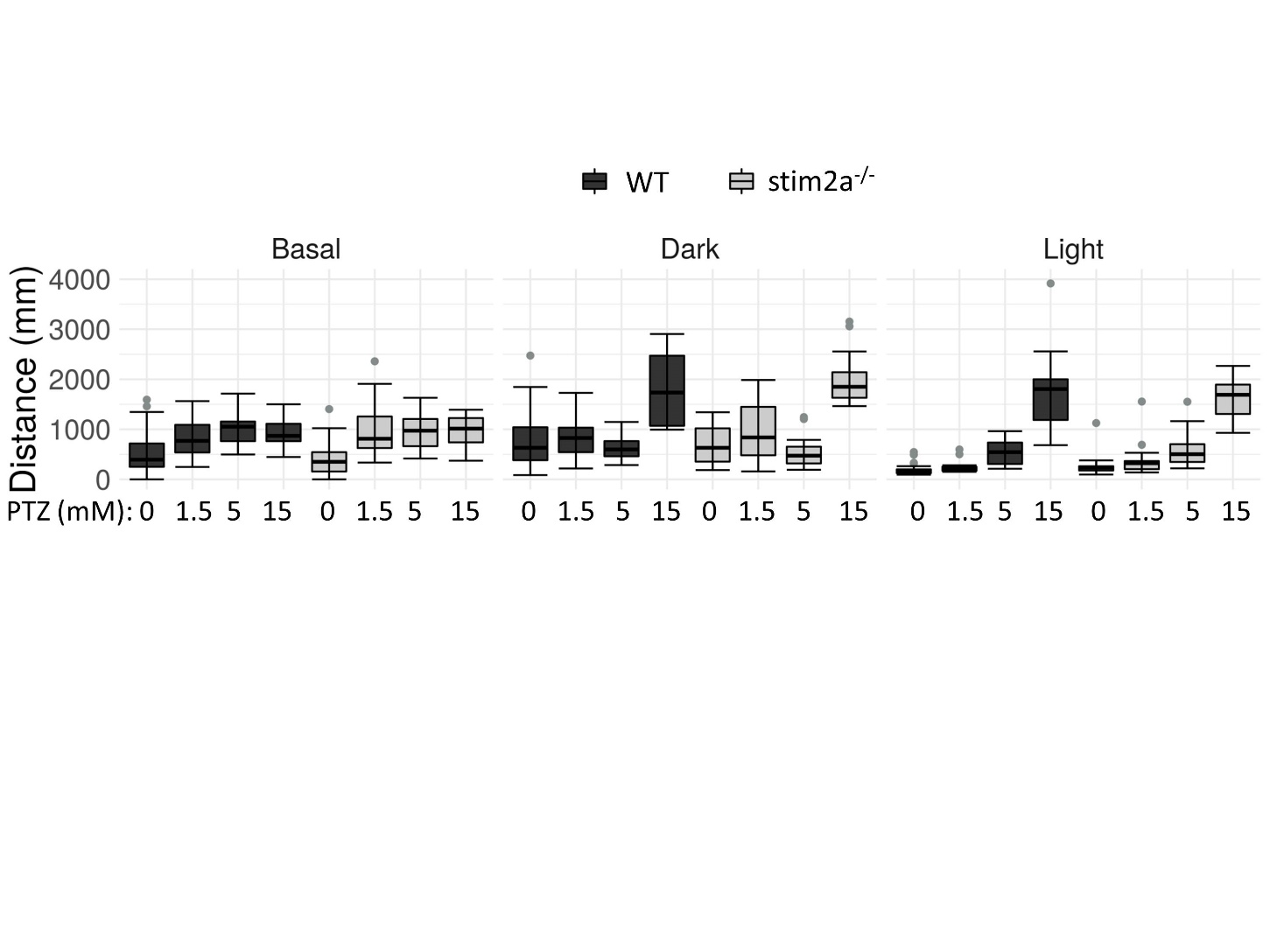


**Fig. S2.** **PTZ treatment:** zero, 1.5, 5, 15 mM PTZ treatment. We opted the higher dose of PTZ from Baraban et. al. [1]. At lower dose of PTZ, we could not detect any differences in WT compared to mutant. However, the highest (15 mM) is known to induce seizure-like behavior in zebrafish larvae starting from 3 dpf [1].

**A B C D**

WT_E3

*stim2a*_E3

WT_Glutamate

*stim2a*_Glutamate

E3 water

E3 water

E3 water

E3 water

E3 water

Glutamate

E3 water

Glutamate

**Fig. S3. Ca fluorescence traces from individual neuron:** Ca fluorescence traces from individual neuron **a**fter automated cell segmentation for Supplementary Video S1, S2, S3 and S4. In each experiment, first 300 sec recording is in E3 medium (indicated by violet dotted arrow) and after that medium was changed (indicated by downward triangle in cyan color and by a dotted line on the Ca fluorescence traces plots) with E3 medium in case of control experiment and with 600 μM in case of treatment (indicated by brown dotted arrow).


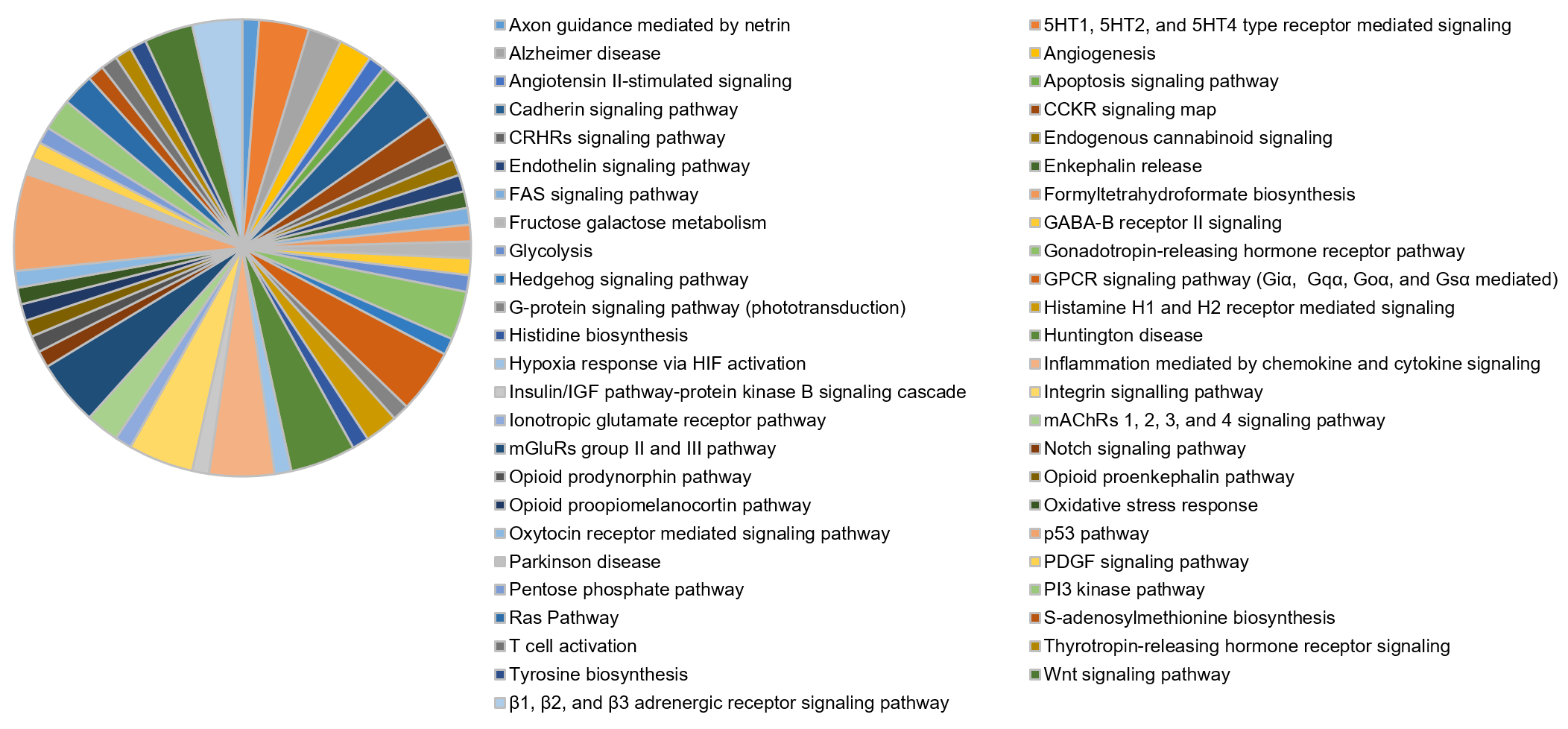


**Fig. S4.** **PANTHER Gene Ontology annotation analysis** [2,3]. The distribution of GO terms was categorized based on PANTHER GO-Slim. Molecular Pathway GO terms (collection of molecular events or operation, with a strict definition of the beginning and end) with 86 pathway hits (297 genes in each annotation).


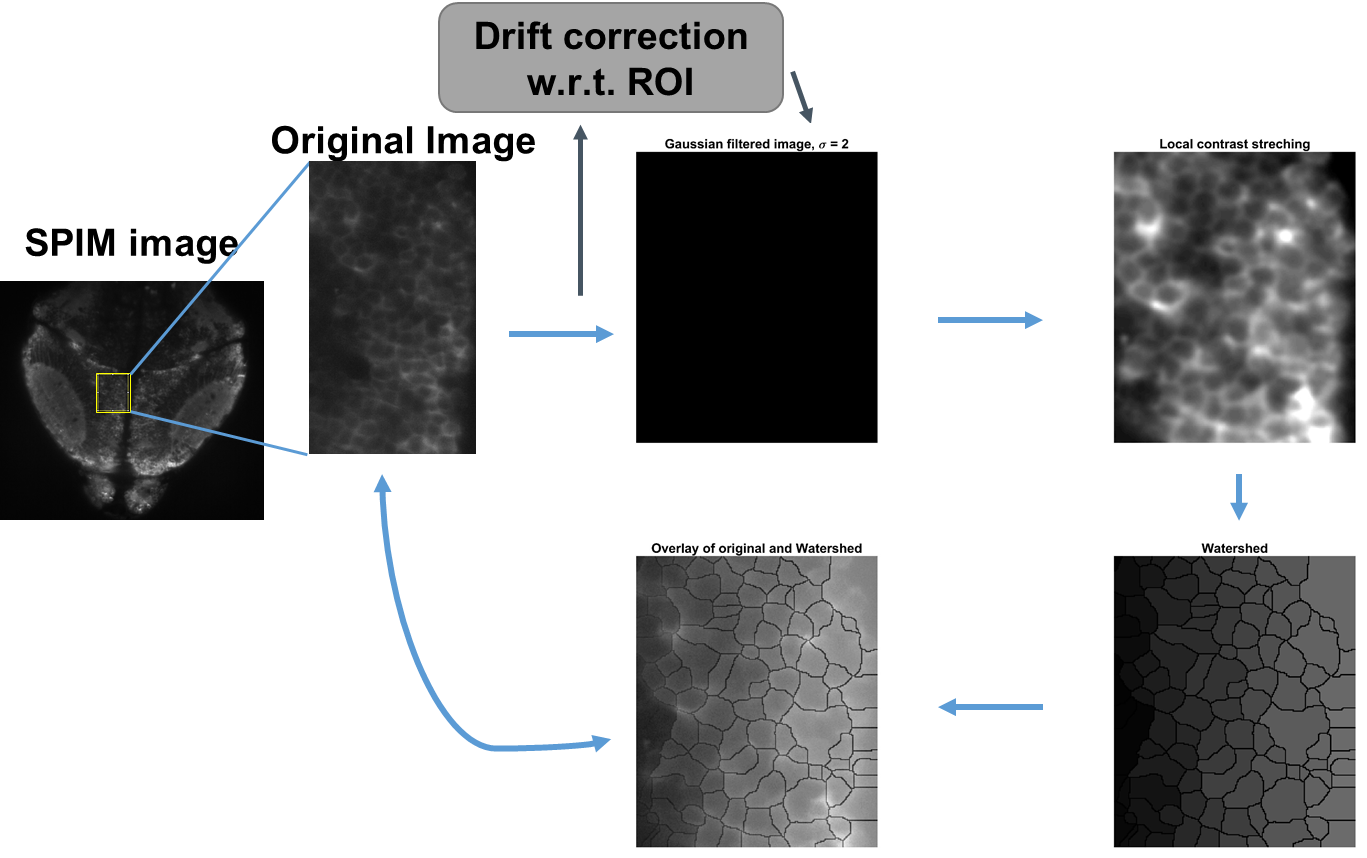


**Fig. S5.** **Automated image segmentation to draw the Region of interest (ROI):** Automated cell segmentation was performed as explained by Panier et. al. [4]

References:

1. Baraban, S.C., et al., Pentylenetetrazole induced changes in zebrafish behavior, neural activity and c-fos expression. *Neuroscience,* **2005**. *131*(3): p. 759-68.
2. Thomas, P.D.; Kejariwal, A.; Campbell, M.J.; Mi, H.; Diemer, K.; Guo, N.; Ladunga, I.; Ulitsky-Lazareva, B.; Muruganujan, A.; Rabkin, S.; et al. PANTHER: A browsable database of gene products organized by biological function, using curated protein family and subfamily classification. *Nucleic Acids Res.* **2003**, *31*, 334–341.
3. Protein Analysis Through Evolutionary Relationships Classification System (PANTHER) Available online: http://www.pantherdb.org (accessed on May 22, 2020).
4. Panier, T., et al., Fast functional imaging of multiple brain regions in intact zebrafish larvae using selective plane illumination microscopy. *Front Neural Circuits,* **2013**. *7*: p. 65.
