# Supplementary material for: Knockout of stim2a Increases Calcium Oscillations in Neurons and Induces Hyperactive-Like Phenotype in Zebrafish Larvae
Source: Int J Mol Sci. 2020 Aug 27;21(17):6198. doi: 10.3390/ijms21176198 (PMC7503814; doi:10.3390/ijms21176198)
Supplement: Supplementary file 1 [file ijms-21-06198-s001.zip › Table S1 stim2a to stim2b comparison.doc]

Table 2

Comparison of features of *stim2a* versus *stim2b* zebrafish knockouts. Differences between zebrafish knockout lines are in bold.

| **Properties and features** | ***stim2a^-/-^* versus WT [this work]** | ***stim2b^-/-^* versus WT** [1] |
| --- | --- | --- |
| Mobility | Higher | Higher |
| Phototaxis | Disrupted/lower | Disrupted/lower |
| Hyperactive behavior | Yes | Yes |
| Thigmotaxis | Increased | Increased |
| Visual motor response | **Bigger, only in light phase** | **Normal** |
| Visual motor response + Glutamate | **No change in mutant**  **Higher in WT** | **Increased mutant mobility** |
| Visual motor response + PTZ | **No change in mutant**  **Higher in WT** | **Dose dependent changes** |
| Calcium signals in OT neurons:   - increase in frequency of oscillations - decrease in oscillation amplitude | Yes  Yes | Yes  Yes |
| Fold of common genes upregulation | *anxa3a* (5.3x), *smc1a* (6.3x ) | *anxa3a* (4.6x), *smc1a* (6.5x ) |
| Expression of different genes:   - upregulated calcium toolkit (CaTK) - downregulated CaTK - upregulated transcription factors (TFs) - downregulated TFs - other up- or downregulated | ***slc25a25b* (4.6x), *pvalb7* (3.9x)*, hpca* (3.6x)*, grinab* (2.9x)*, mast2* (2.7x)**  ***pkn3* (-2.7x), *hp* (-2.6x)*, stim2a* (-2.5x)**  ***stat6* (3.6x), *klf2a* (3.5x)*, prdm2a* (2.7x)*, fosl1a* (2.5x)*, sgf29* (2.1x)*,***  ***fosab* (-2.8x), *npas4l* (-2.7x)*, nr0b2a* (-2.4x), *egr4* (-2.4x)*,***  ***scin* (3.7x), *mmp13a* (3.5x), *vwa2* (2.8x), *dgkaa* (2.5x), *cdh13* (2.2x)** | ***anxa1c* (2.1x), *anxa5a* (1.6x)**  ***homer2* (-2x)**  ***ecrg4b* (3.7x), *gpr39* (2.9x)**  ***rrm2* (-5x), *ngdn* (-2x), *per1a* (-2x)** |

1.	Wasilewska, I.; Gupta, R.K.; Palchevska, O.; Kuźnicki, J. Identification of Zebrafish Calcium Toolkit Genes and their Expression in the Brain*. Genes (Basel*)**. 20**19*,* 10, 230.
